# A Dipyrrin Programmed for Covalent Loading with Fullerenes

**DOI:** 10.1002/chem.201801995

**Published:** 2018-06-19

**Authors:** Chengjie Li, Klaus Wurst, Bernhard Kräutler

**Affiliations:** ^1^ Institute of Organic Chemistry and Centre of Molecular Biosciences University of Innsbruck Innrain 80/82 6020 Innsbruck Austria; ^2^ Key Laboratory for Advanced Materials and Institute of Fine Chemicals School of Chemistry & Molecular Engineering East China University of Science & Technology Meilong Rd 130 200237 Shanghai China; ^3^ Institute of General, Inorganic & Theoretical Chemistry University of Innsbruck Innrain 80/82 6020 Innsbruck Austria

**Keywords:** C_60_-fullerene, cheletropic reaction, cycloaddition, Diels–Alder reactions, porphyrins

## Abstract

We describe here a di‐(β,β′‐sulfoleno)pyrrin programmed for efficient and specific β,β′‐functionalization via [4+2] cycloaddition reactions. At 120 °C and in the presence of an excess of C_60_‐fullerene the di‐(β,β′‐sulfoleno)pyrrin decomposed cleanly, furnishing a di‐(β,β′‐fullereno)pyrrin and the corresponding monofullereno‐dipyrrin in an overall yield of 96 %. Hence, relatively mild thermolysis of the di‐(β,β′‐sulfoleno)pyrrin induced stepwise extrusion of two equivalents of SO_2_, producing highly reactive dipyrrin‐β,β′‐diene intermediates readily, providing a very effective path to [4+2]‐cycloadducts. As presented here by the example of the covalent attachment of C_60_‐fullerene units, a convenient general methodology for the efficient synthesis of covalent dipyrrin β,β′‐cycloadducts is made available.

2,2′‐Dipyrrins contain two conjugated pyrrolic rings,^1^ and are useful building blocks for the synthesis of porphyrins and related tetrapyrrolic macrocycles.^2^ Dipyrrins have also attracted considerable attention, in their own right, due to their unique photo‐physical and electrochemical properties, which are modified in interesting ways after coordination with transition metal ions, or with boron in BODIPYs.^1^, 2b, 3 Various methods have been developed, in order to introduce functional groups at the pyrrole units of 2,2′‐dipyrrins to tune their properties for a variety of applications, for example, as photo‐ and redox‐active compounds,^4^ as probes in bio‐imaging,^5^ in energy up‐conversion materials,^6^ and as microstructure building blocks.^7^ The modification of 2,2′‐dipyrrins occurs with ease at their pyrrole‐*α* positions, and the selective introduction of substituents at the pyrrole‐β position has also been worked out.^8^ In contrast, incorporation of ring‐fused groups on the pyrrole units of dipyrrins is still a demanding task.^9^ However, the preparation of β,β′‐sulfolenopyrroles has opened access to highly reactive pyrrole‐2,3‐dienes^10^ suitable for [4+2] cycloaddition of various dienophiles at the pyrrole β,β′‐positions.^11^

We have recently designed ‘programmed“ porphyrinoid compounds, such as the symmetric tetra‐(β,β′‐sulfoleno)porphyrins (see Scheme 1), for the purpose of the synthesis of novel porphyrinoid assemblies.10b, 12 From tetra‐(β,β′‐sulfoleno)porphyrins four SO_2_ units were readily extruded consecutively at 140 °C, generating an array of highly reactive porphyrin‐dienes, which provided a unique basis for attaching various dienophiles.12b, 12c In contrast, the corresponding, less symmetric tetra‐(β,β′‐sulfoleno)corrole^13^ liberated the diene units quickly at the directly linked (‘western′) pyrroles (9 min at 140 °C) and allowed the regioselective loading of a corrole with two C_60_‐fullerene units at the ”western“ pyrroles. Broadly functioning paths to porphyrinoid conjugates with C_60_‐fullerenes are very useful, as these furnish attractive components of photovoltaic and other materials.^14^

**Scheme 1:**
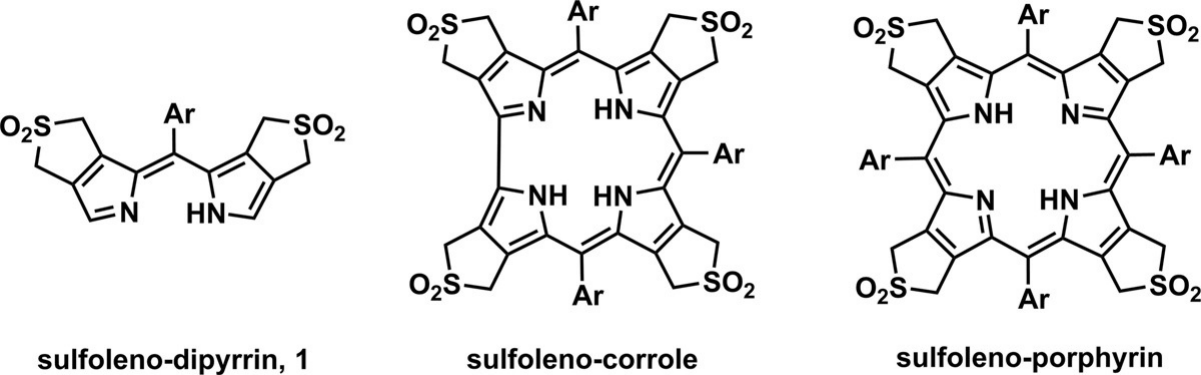
Structural formulae of the β,β′‐sulfolenopyrrole based dipyrrin **1**,^15^ of a tetrasulfolenocorrole^13^ and a symmetrical tetrasulfolenoporphyrin.12a, 12b.

As reported here, heating of the di‐(β,β′‐sulfoleno)‐dipyrrin **1** 15 liberated SO_2_ very readily (in contrast to the analogous β,β′‐sulfolenopyrroles11a, 16), providing a mild and efficient path for twofold [4+2]‐cycloaddition of the dienophile C_60_.

Single crystals of the diene‐masked dipyrrin di‐(β,β′‐sulfoleno)pyrrin **1** 15 (monoclinic space group *P*2_1_/*c*) were obtained by mixing of *n*‐hexane into the solution of **1** in CH_2_Cl_2_. The unit‐cell contained four molecules of **1**. In the conjugated π‐system of **1** the individual bonds showed significant bond‐length alteration (Figure 1), as found in other dipyrrins,^17^ and consistent with the formula shown in Scheme 2. The methine bridge of **1** exhibits *Z*,*cis*‐geometry and an H‐bond is observed in the crystal between H2N to N1. The *Z*,*cis*‐geometry is a common structural feature of 2,2′‐dipyrrins, when not doubly protonated or attached to metal‐ions with specific binding modes.^18^ The best planes through the two pyrrolic rings in the conjugated system were roughly co‐planar (3.5° dihedral angle). The mean planes of the aryl group (at the dipyrrin *meso*‐position) and of the dipyrrin core were at a dihedral angle of 74.1°, similar to the situation in other *meso*‐phenyl substituted dipyrrins.^17^, 18b, 19 The angle C4‐C5‐C6 (124.2°, see Figure S1) of the di‐(β,β′‐sulfoleno)pyrrin **1** is only slightly smaller than that (127.2°) in the (2:1)‐complex of Zn^II^‐ions with **1**,^15^ and similar to that of a related dipyrrin with an aromatic *meso*‐substituent.^20^ The two S atoms are positioned on opposite sides with respect to the plane of the dipyrrin core, with out of plane distances of 0.16 and 0.28 Å for S1 and S2, respectively. In addition, the crystal structure revealed a remarkable π‐stacking arrangement of the 2,2′‐dipyrrin **1**. In neighbors, tight π–π packing of the dipyrrin cores of two molecules of **1** oriented the planes of the conjugated π‐system in parallel, and at a mutual distance of 3.75 Å. However, the two molecules of **1** are related by a center of inversion in a dimer, so that their dipyrrins‐units are pointing into opposite directions (see Figure 1).

**Figure 1:**
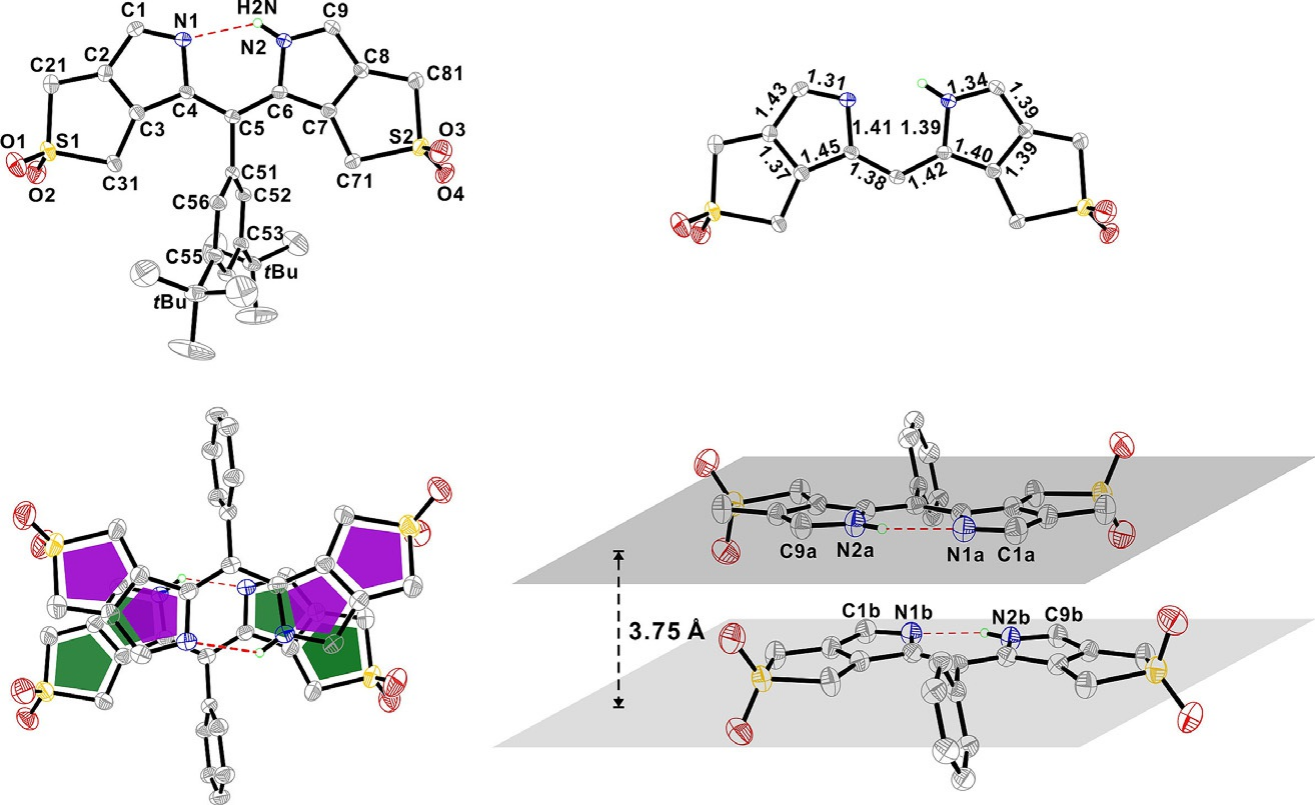
ORTEP plot of the crystal structure of the di‐(β,β′‐sulfoleno)pyrrin **1**. Top left: front view with numbering system; H‐atoms at carbons were omitted for the sake of clarity. Top right: front view and bond lengths of the dipyrrin core of **1**; H‐atoms at carbons and the 3,5‐di‐*tert*‐butyl‐phenyl group were omitted for sake of clarity. Bottom left: top view of π‐stacked dimer, highlighting the dipyrrin cores. Bottom right: side view of two π‐stacked dipyrrins **1**, highlighting the distance between the two dipyrrin planes. In both figures at the bottom H‐atoms at carbons and the *tert*‐butyl groups were again omitted.

**Scheme 2:**
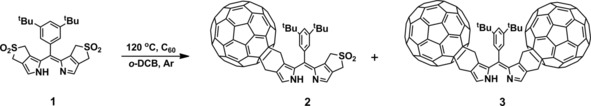
Thermolysis of dipyrrin **1** in the presence of C_60_ resulted in the loss of SO_2_ and the covalent attachment of C_60_‐fullerene units to the β‐ and β′‐positions of the pyrrole moieties.

Heating of a deoxygenated solution of 2.33 mg (4.55 μmol) of di‐(β,β′‐sulfoleno)pyrrin **1** and 23.45 mg (32.6 μmol) of C_60_ in 2 mL of 1,2‐dichlorobenzene (*o*‐DCB) at 120 °C for 20 min resulted in the complete decomposition of **1** (Scheme 2). From the reaction mixture 1.3 mg (or 24 % yield) of the monofullereno‐dipyrrin **2** and 6.0 mg (or 72 % yield) of bisfullereno‐dipyrrin **3** were isolated after column chromatography and crystallization from CS_2_/EtOH.

The UV/Vis spectrum of the yellow 2.2′‐dipyrrin **1** exhibits the typical absorption maximum at 435 nm,^15^ as is shown in Figure 2. Upon attachment of C_60_‐fullerene units, the absorption maximum shifted by 18 nm to longer wavelength for the monoadduct **2**, and by 20 nm in the spectrum of the bisadduct **3**. The dihydrofullerene addends cause additional absorptions, with very characteristic weak maxima at 702 nm^21^ and broad bands with increasing intensity at shorter wavelengths, consistent with one or two C_60_‐addends in **2** and **3**, respectively. Similar effects on the UV/Vis‐spectral characteristics were found in covalent porphyrin β,β′‐conjugates of C_60_, which were obtained by thermolysis of tetrasulfoleno‐porphyrin.12b, 13 Fluorescence, as reported for **1** and its Zn‐complex,^15^ is absent in the fullerene adducts **2** and **3**, where the dipyrrin luminescence is quenched effectively by the closely positioned C_60_‐addends.

**Figure 2:**
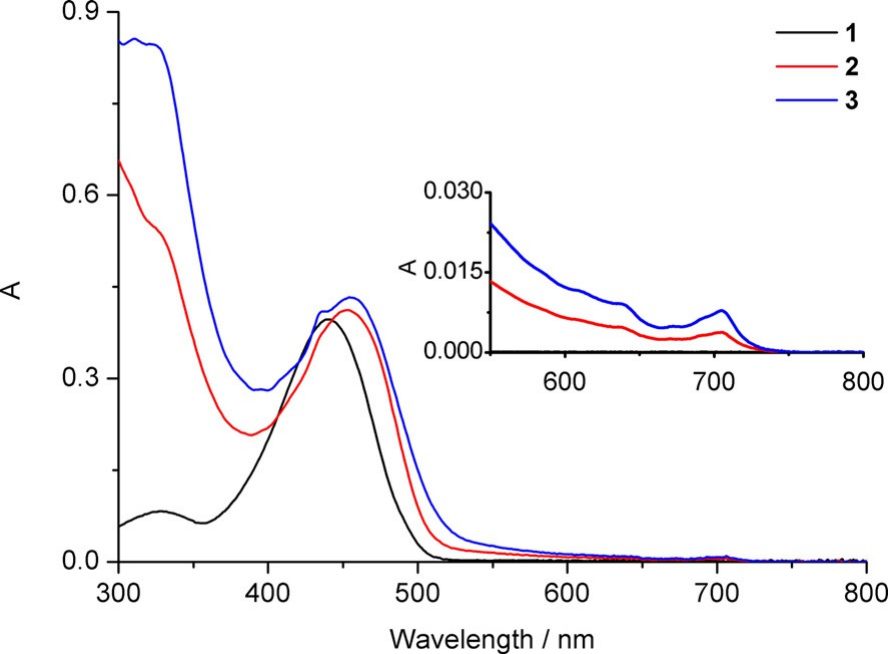
UV/Vis spectra of 2,2′‐dipyrrin **1** (black trace) and of its mono‐fullereno and bisfullereno‐derivatives **2** (red trace) and **3** (blue trace), dissolved in toluene (*c*=1.53×10^−5^ m).

The molecular formulas of fullereno‐dipyrrins **2** and **3** were deduced from MALDI‐TOF mass spectra. The spectrum of **2** featured a pseudo‐molecular ion at *m*/*z* 1169.0 [*M*+H]^+^, corresponding to C_87_H_32_N_2_O_2_S. A strong fragment at *m*/*z* 1105.2 [*M*−SO_2_+H]^+^ indicated the loss of a SO_2_ group. The mass spectrum of **3** confirmed its molecular formula as C_147_H_32_N_2_ by showing a molecular ion at *m*/*z=*1824.6 [*M*]^+^ and a strong fragment at *m*/*z* 1105.2, from loss of one fullerene unit.

The structures of the fullereno‐dipyrrins were established by detailed analysis with one and two‐dimensional NMR spectra (^1^H,^1^H‐COSY and ROESY, ^1^H,^13^C‐HSQC and HMBC spectra). The monoadduct **2** is less symmetric than its precursor **1**, and its NMR spectra showed, correspondingly, more signals (see Figures 3 and 4). In the ^1^H NMR spectrum of **2**, for example, four singlets of methylene groups were seen at intermediate field, and two singlets for α‐pyrrolic protons at low field (see Figure 4). In the spectrum of **2**, the two methylene singlets at 3.14 ppm and 4.10 ppm were present at a position quite similar to that in the ^1^H NMR spectrum of **1**. Two further methylene singlets in the spectrum of **2** appeared at 3.60 ppm (broad singlet) and 4.46 ppm, shifted to lower field by de‐shielding by the close‐by fullerene unit. Each of these latter methylene signals coupled to C‐atoms with chemical shift values of 39.2 ppm and 38.3 ppm (as seen in HSQC‐spectra), as is typical for pyrrole β‐methylene groups linked to a fullerene (Table S1 and Figure S5).^13^ In contrast, the other two methylene signals (3.14 ppm and 4.10 ppm) correlated to carbons at 55.5 ppm and 53.4 ppm in the HSQC spectra of **2**, indicating their attachment to the remaining sulfolene unit (Figure S5). The deduced location of methylene groups was confirmed in a ^1^H,^1^H‐ROESY spectrum. There, the signals at 3.60 and 3.14 ppm showed correlations with H‐atoms at the *ortho*‐position of the *meso*‐phenyl group and were assigned to H_2_C3^1^ and H_2_C7^1^, respectively (Figure 3). Such NOE correlations were not seen for the two signals at 4.46 and 4.10 ppm, consistent with assignment as H_2_C2^1^ and H_2_C8^1^, respectively. The methylene groups exhibited a single resonance, each, in the ^1^H NMR spectrum of **2** at room temperature, due to rapid conformational inversion of the six‐membered ring connecting the fullerene and pyrrole units of **2**, as has been observed in analogous fullereno‐porphyrins.12b

**Figure 3:**
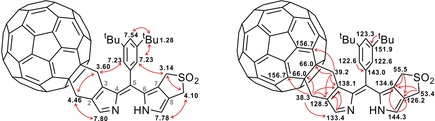
^1^H–^1^H correlations observed in ^1^H,^1^H‐ROESY (left, atom numbering is given in gray) and ^1^H–^13^C correlations from ^1^H,^13^C‐HSQC and HMBC spectra (right) of a solution of **2** in CDCl_3_/CS_2_ (500 MHz, 25 °C).

**Figure 4:**
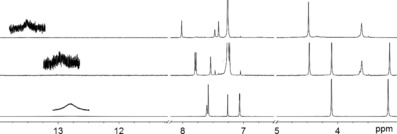
Relevant sections of ^1^H NMR spectra of the fullerene bis‐adduct **3** (top, CDCl_3_/CS_2_, 500 MHz), of the fullerene mono‐adduct **2** (center, CDCl_3_/CS_2_, 500 MHz) and of the dipyrrin **1** (bottom, in CDCl_3_, 300 MHz).

A singlet at 7.80 ppm was assigned to HC1, as it correlated with the fullerene‐linked methylene group at 4.46 ppm in the ROESY spectrum of **2** (Figure 3). The other low field singlet (at 7.78 ppm) was assigned to HC9, as it correlated with the methylene group at 4.10 ppm (Figure 3). The attachment of a C_60_‐fullerene was further secured by a ^1^H,^13^C‐HMBC spectrum, in which the methylene signals at 4.46 ppm and 3.60 ppm correlated with ^13^C‐signals at *δ*=133.4 ppm (pyrrole C1), as well as at *δ*=66.0 ppm (*sp* ^*3*^‐C of the fullerene unit) and at 156.7 ppm (adjacent *sp* ^*2*^‐C of the fullerene), that is, the typical chemical shift values of C‐atoms at the fullerene C[6,6]‐bond that has undergone [4+2] cycloaddition (Figure S6).12b, 13, 21, 22 The pyrrole NH gave a broad weak signal at slightly lower field, when compared to the spectrum of its precursor **1**.

The di‐(β,β′‐fullereno)pyrrin **3** exhibited a simpler pattern of the signals in its ^1^H NMR spectrum than the monoadduct **2**, due to its more symmetric effective structure. Only two (broad) singlets of the eight diastereotopic methylene protons appeared at 4.48 ppm and 3.60 ppm as a consequence of fast conformational equilibration. The pyrrole α‐H was shifted to 8.02 ppm (Figure 3 and Table S1 in the Supporting Information). In the ^1^H,^13^C‐HSQC spectrum of **3**, the two methylene group singlets at 3.60 ppm and 4.48 ppm of the correlated to carbon resonances at 38.6 and 40.0 ppm of the neighboring fullerene addend (Figure S7), and, in a ^1^H,^13^C‐HMBC spectrum, to the typical quaternary carbon of the fullerene moiety at 66.4 ppm (Figure S8). These correlations secured the attachment of the two fullerene units in **3**. The shielding effect of the phenyl group at the dipyrrin *meso*‐position assisted the assignment of the signal at 3.60 ppm to H_2_C3^1^ and H_2_C7^1^. When the ^1^H NMR data of **1**, **2** and **3** are compared, the signals of the pyrrole NH and α‐H′s, of the protons of the methylene group next to fullerene and at the *ortho*‐position of the *meso*‐phenyl substituent shift to lower field, upon consecutive loading with the C_60_‐fullerene, whereas the signal of the proton in the *para*‐position of the *meso*‐phenyl group shifts to higher field (Figure 3 and Figure S2).

Single crystals of the di‐(β,β′‐fullereno)pyrrin **3** grew from a solution of **3** in CS_2_ when EtOH was mixed in slowly at 23 °C. Bis‐adduct **3** crystalized in the monoclinic space group *C*2/*c*. The unit‐cell contained four molecules of **3** and half a molecule in the asymmetric unit, which was completed by a crystallographic twofold rotation axis. The NH in the dipyrrin unit was disordered due to the effective symmetry of the structure of the dipyrrin core.

In **3** the two fullerene units are positioned on opposite faces with respect to the reference plane of the dipyrrin core (see Figure 5). The two pyrrolic rings in the dipyrrin core are twisted with a dihedral angle of 9.5° in the bisadduct **3** (increased from 3.5° in **1**). The angle between the mean planes of the aryl group and of the dipyrrin core has increased to 82.8°, a consequence of the steric bulk of the C_60_ substituent. In the crystal, the mean planes of the dipyrrin cores of neighboring molecules are positioned in parallel and at a distance of 10.03 Å. Two neighbor molecules relate to each other by a center of inversion, so that their dipyrrin cores are oriented in opposite directions. The fullerene units of **3** prevent intermolecular π–π stacking interactions of the dipyrrin core. They make remarkably short intermolecular contacts with closest distances between C7−C49c of 3.28 Å and C27−C36b of 3.36 Å (edge‐to‐edge interactions in the same row or column) (see Figure 5 and Figure S3).

**Figure 5:**
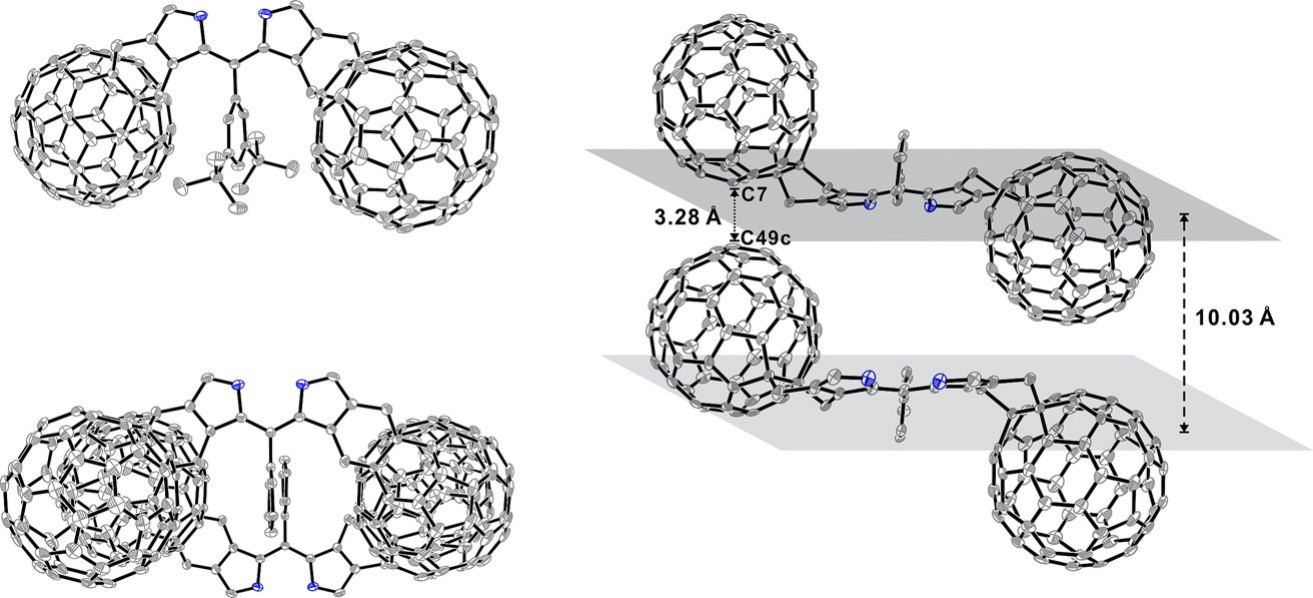
ORTEP plot of the crystal structure of the di‐(β,β′‐fullereno)pyrrin **3**. Top left: front view, in which H‐atoms were omitted for the sake of clarity; bottom left: top view of π‐stacked neighbors that relate by a center of inversion (with H‐atoms and *tert*‐butyl groups omitted). Right: side view of π‐stacked neighbors, highlighting crucial distances between the planes of the two dipyrrin cores and between next neighbor carbon atoms of fullerene moieties.

In conclusion, the SO_2_ groups of the di‐(β,β′‐sulfoleno)pyrrin **1** are rapidly extruded consecutively at 120 °C. This furnishes reactive diene intermediates that can be trapped by the C_60_‐fullerene via rapid [4+2] cycloaddition, attaching C_60_ units at the pyrrole β,β′‐positions of the dipyrrin core and furnishing the bisadduct **3** as final product (Scheme 3). The intermediate mono(β,β′‐fullereno)‐adduct **2** still harbors one sulfolene function, which is available for further thermal diene formation and subsequent [4+2] cycloaddition reaction with alternative dienophiles. Conjugates of fullerenes with dipyrrins represent attractive photo‐ and redox‐active components,^1^, 4b as has also been established for various of their porphyrinoid analogues.^14^, ^23^

**Scheme 3:**
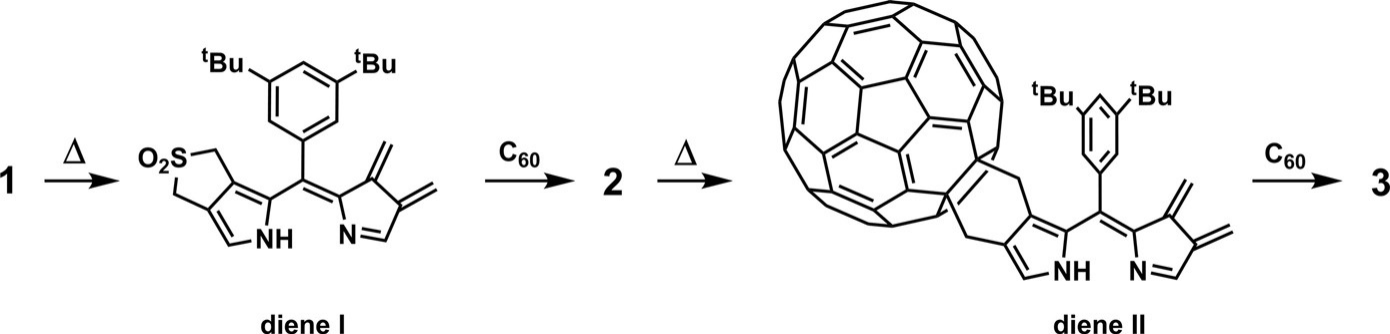
Thermolysis of di‐(β,β′‐sulfoleno)pyrrin **1** in the presence of C_60_‐fullerene generates the reactive diene intermediates I and II, which undergo covalent attachment of C_60_‐units by sequential [4+2]‐cycloaddition, furnishing the di‐(β,β′‐fullereno)pyrrin **3**.

The di‐(β,β′‐sulfoleno)pyrrin **1** serves as a masked form of a highly reactive bis‐diene, undergoing [4+2]‐cycloaddition reactions readily with typical dienophiles. In earlier work, symmetric tetra‐(β,β′‐sulfoleno)porphyrins and a tetra‐(β,β′‐sulfoleno)corrole were used as reactive precursors ‘programmed“ for loading with C_60_‐fullerene12b, 12d, 13 and benzoquinone12c by [4+2] cycloaddition. However, the three β,β′‐sulfolenopyrrole derivatives with extended conjugated systems differ strongly by the ease and regio‐selectivity of the loss of their SO_2_ moieties. Unlike the situation found here with the dipyrrin **1** (rapid conversion at 120 °C), the detachment of two SO_2_ moieties from the tetra‐(β,β′‐sulfoleno)porphyrin required longer heating at 140 °C,12b and the corresponding conversion of analogous β,β′‐sulfolenopyrroles even needed prolonged heating at 210 °C.11a,11c

In the present study, the representative di‐(β,β′‐sulfoleno)pyrrin **1** was shown to undergo a thermally induced process readily that set the stage for [4+2]‐cycloaddition of the C_60_‐fullerene (or, presumably, of other fullerenes^24^). Use of still other dienophiles, such as quinones,12c will provide the opportunity for producing dipyrrins with further extended chromophoric systems. Indeed, the di‐(β,β′‐sulfoleno)pyrrin **1** is ‘programmed“ for heat‐induced loss of SO_2_ and subsequent introduction of a variety of covalent modifications at the pyrrole β‐positions by [4+2]‐cycloaddition chemistry. As was similarly explored with tetra‐(β,β′‐sulfoleno)porphyrins12b–12e and a tetra‐(β,β′‐sulfoleno)‐corrole,^13^ the reactive dipyrrin **1** proofed to be a convenient 2.2′‐dipyrrin building block. 2,2′‐Dipyrrins are versatile bidentate ligands for coordination of metal ions^1^, ^15^ and for higher order supramolecular architectures,^1^ useful in imaging and energy conversion. Indeed, the di‐(β,β′‐sulfoleno)dipyrrin **1** was first studied as a model component in zinc chelates,^15^ as models of the structurally more complex transition metal complexes of natural yellow bilins, such as phylloxanthobilins^25^ and bilirubin.^26^ The ”programmed“ di‐(β,β′‐sulfoleno)pyrin **1** is a novel member of semi‐rigid pyrrole‐based compounds, helping to expand the recently developed ‘porphyrin LEGO′ methodology12d to the assembly of corresponding covalent molecular constructs in the fields of 2,2′‐dipyrrins and BODIPYs.^1^, ^2^, ^3^

## Experimental Section

*General*: Dichloromethane (CH_2_Cl_2_) was from Sigma–Aldrich (Steinheim, Germany), and was distilled and filtered over basic Alox before use. Toluene, *o*‐dichlorobenzene (*o*‐DCB), carbon disulfide (CS_2_), ethanol (EtOH), ethyl acetate (EtOAc) and *n*‐hexane (*n*‐C_6_H_14_) were from VMR (Leuven, Belgium); C_60_ was purchased from Merck Corporation (USA). Column chromatography (CC): Fluka silica gel 60 (230≈400 mesh). Thin layer chromatography (TLC): Merck 0.25 mm silica gel 60 plates without GF254. Equipment: UV/Vis: Agilent Cary 60 UV/Visible, *λ*_max_ in nm (log *ϵ*). Fluorescence (FL): Varian Cary Eclipse, *λ* in nm (rel. intensity); Nuclear magnetic resonance (NMR) spectra: Bruker 300 or Varian 500 Unity plus at 298 K, chemical shifts (*δ*) in ppm, with ^1^H NMR: *δ* (CHCl_3_)=7.26 ppm, ^13^C‐NMR: *δ* (CDCl_3_)=77.16 ppm. atom numbering as for X‐ray data (see Figures 1 & 5). MALDI‐TOF: Bruker Daltonics Ultraflex with *trans*‐2‐[3‐(4‐*tert*‐butylphenyl)‐2‐methyl‐2‐propenylidene]‐malononitrile as matrix.

*Synthesis*: A solution of di‐(β,β′‐sulfoleno)pyrrin (**1**)^15^ (2.33 mg, 4.55 μmol) and C_60_ (23.45 mg, 32.57 μmol, 7.2 moleq) in 2 mL *o*‐dichlorobenzene (*o*‐DCB) was purged with Ar for 5 minutes and heated at 120 °C for 20 minutes under Ar in the dark. The reaction mixture was diluted with 1 mL *n*‐C_6_H_14_ and loaded on silica gel column (2 cm×15 cm). Bisadduct **3** was washed down with toluene and monoadduct **2** was eluted by toluene with 5 % of EtOAc. After drying under reduced pressure, 1.3 mg of brown solid **2** were obtained (24 % yield) after precipitation in CS_2_/EtOH, and 6.0 mg of **3** were obtained as brown single crystals (72 % yield) by crystallization in CS_2_/EtOH (2:3, v/v). Monoadduct **2** UV/Vis (toluene): *λ*_max_ (log *ϵ*): 330 (4.54), 434.5sh (4.38), 453.5 (4.43), 702 (2.50). Monoadduct **2** showed no fluorescence emission. ^1^H NMR (500 MHz, 25 °C, 1.7×10^−3^ m in CDCl_3_/CS_2_=0.4 mL/0.1 mL): 1.28 (s, *t*Bu), 3.14 (s, H_2_C‐7^1^), 3.60 (s, H_2_C‐3^1^), 4.10 (s, H_2_C‐8^1^), 4.46 (s, H_2_C‐2^1^), 7.23 (d, *J=*1.3 Hz, HC‐5^2^ and HC‐5^6^), 7.54 (t, *J=*1.3 Hz, HC‐5^4^), 7.78 (s, HC‐9), 7.80 (s, HC‐1), 12.96 (br s, HN‐2). MALDI‐TOF MS: 1171.0 (10), 1170.0 (19), 1169.0 (23, [*M*+H]^+^ Calcd. for [*M*+H]^+^ C_87_H_33_N_2_O_2_S *m*/*z=*1169.2); 1107.1 (24), 1106.1 (71), 1105.2 (100, [*M*−SO_2_+H]^+^). Bisadduct **3** UV/Vis (toluene): *λ*_max_ (log *ϵ*): 327 (4.74), 435 (4.43), 455 (4.45), 702 (2.70). Bisadduct **3** showed no fluorescence emission. ^1^H NMR (500 MHz, 25 °C, 1.0×10^−3^ m in CDCl_3_/ CS_2_=3.5/1.5): 1.18 (s, *t*Bu), 3.60 (s, H_2_C‐3^1^ and H_2_C‐7^1^), 4.48 (s, H_2_C‐2^1^ and H2C‐8^1^), 7.41 (d, *J=*1.4 Hz, HC‐5^2^ and HC‐5^6^), 7.47 (t, *J=*1.4 Hz, HC‐5^4^), 8.02 (s, HC‐9 and HC‐1), 13.56 (br. s, HN‐2). MALDI‐TOF MS: 1828.5 (2), 1827.6 (7), 1826.6 (14), 1825.6 (22), 1824.6 (10, [*M*]^+^ Calcd. for [*M*]^+^ C_147_H_32_N_2_ *m*/*z=*1824.3); 1107.2 (30), 1106.2 (79), 1105.2 (100, [*M*−C_60_+H]^+^).

*Crystallographic data*: X‐ray analyses: data collection on a Nonius Kappa CCD (for **1**) and on a Bruker D8 QUEST (for **3**), both equipped with graphite mono‐chromatized Mo‐Kα‐radiation (*λ*=0.71073 Å). https://summary.ccdc.cam.ac.uk/structure-summary?doi=10.1002/chem.201801995 1837889 (**1**) and 1837890 (**3**) (excluding structure factors) contain the supplementary crystallographic data for this paper. These data are provided free of charge by http://www.ccdc.cam.ac.uk/.

## Conflict of interest

The authors declare no conflict of interest.

## Supporting information

As a service to our authors and readers, this journal provides supporting information supplied by the authors. Such materials are peer reviewed and may be re‐organized for online delivery, but are not copy‐edited or typeset. Technical support issues arising from supporting information (other than missing files) should be addressed to the authors.

Supplementary
