# Supplementary material for: A Dipyrrin Programmed for Covalent Loading with Fullerenes
Source: Chemistry. 2018 Jun 19;24(40):10032–7. doi: 10.1002/chem.201801995 (PMC6099335; doi:10.1002/chem.201801995)
Supplement: Supplementary file 1 — Supplementary [file CHEM-24-10032-s001.pdf]

CHEMISTRY
A European Journal

Supporting Information

A Dipyrrin Programmed for Covalent Loading with Fullerenes

Chengjie Li,[a, b] Klaus Wurst,[c] and Bernhard Kräutler*[a]

chem_201801995_sm_miscellaneous_information.pdf

Supporting Information

A Dipyrrin Programmed for Covalent Loading with Fullerenes

Chengjie Li [1,2], Wurst Klaus [3], Bernhard Kräutler [1,*]

1. Institute of Organic Chemistry & Centre of Molecular Biosciences, University of Innsbruck, A-6020 Innsbruck, Austria

2. Key Laboratory for Advanced Materials and Institute of Fine Chemicals, School of Chemistry & Molecular Engineering, East China University of Science & Technology, Meilong Rd 130, 200237 Shanghai, China

3. Institute of General, Inorganic & Theoretical Chemistry, University of Innsbruck, A-6020 Innsbruck, Austria

General.

Nuclear magnetic resonance (NMR) spectra: Bruker 300 or Varian 500 Unity plus at 298 K, chemical shifts (δ) in ppm, with ^{1}H-NMR: δ ($CHCl_3$) = 7.26 ppm and ^{13}C-NMR (^{1}H,^{13}C-HSQC and HMBC): δ ($CDCl_3$) = 77.16 ppm. The chemical shifts of the Hs or Cs were assigned on basis of ^{1}H,^{1}H or ^{1}H,^{13}C correlations 2D NMR spectra (^{1}H,^{1}H-COSY and ROESY, ^{1}H,^{13}C-HSQC and HMBC).

See main part for other experimental details.

Table S1. ^{1}H- and ^{13}C-NMR chemical shift data of dipyrrin **1** ($CDCl_3$, 300 MHz), monoadduct **2** ($CDCl_3/CS_2$, 500 MHz) and bisadduct **3** ($CDCl_3/CS_2$, 500 MHz) (see Figure S1 for numbering of atoms of the dipyrrin core).

	Dipyrrin **1**	Monoadduct **2**		Bisadduct **3**	
	^{1}H	^{1}H	^{13}C	^{1}H	^{13}C
C1	7.58	7.80	133.4	8.02	140.1
C2			128.5		131.1
C2^{1}	4.10	4.46	38.3	4.48	38.6
C3			138.1		137.9
C3^{1}	3.17	3.60	39.2	3.60	40.0
C51			143.0		142.3
C52 / C56	7.06(d, 1.7) [a]	7.23(d, 1.5) [a]	122.6	7.41(d, 1.5) [a]	123.3
C53			151.9		151.4
C54	7.61(t, 1.7) [a]	7.54(t, 1.5) [a]	123.3	7.47(t, 1.5) [a]	123.4
C7			134.6		137.9
C7^{1}	3.17	3.14	55.5	3.60	40.0
C8			126.2		131.1
C8^{1}	4.10	4.10	53.4	4.48	38.6
C9	7.58	7.78	144.3	8.02	140.1
H2N	12.76	12.96		13.56	
tBu	1.35	1.28	31.3	1.18	31.2
tBuC			34.8		34.8
C_{60}[C1F, C6F]			66.0		66.4
C_{60}[C2F,C5F,C7F, C10F]			156.7		157.2

[a] chemical shift value (signal type, coupling constant) for protons of the di-tert-butylphenyl group

Table S2. Crystal data and structure refinement for 2,2'-dipyrrin **1**.

Empirical formula	$C_{27}H_{32}N_2O_4S_2 \times CH_2Cl_2$	
Formula weight	597.59	
Space group	$P2_1/c$	
Unit cell dimensions	a = 14.6789(3) Å	α= 90°.
	b = 12.9361(3) Å	β= 114.632(2)°.
	c = 17.0038(2) Å	γ = 90°.
Volume	2935.00(11) $Å^3$	
Z	4	
Density (calculated)	1.352 Mg/m^3	
Absorption coefficient	0.400 mm^{-1}	
F(000)	1256	
Crystal size	0.3 x 0.2 x 0.1 mm^3	
Theta range for data collection	2.053 to 24.999°.	
Index ranges	-17<=h<=17, -15<=k<=15, -20<=l<=17	
Reflections collected	18505	
Independent reflections	5166 [R(int) = 0.0232]	
Completeness to theta = 24.999°	99.7 %	
Absorption correction	None	
Refinement method	Full-matrix least-squares on F^2	
Data / restraints / parameters	5166 / 1 / 347	
Goodness-of-fit on F^2	1.035	
Final R indices [I>2sigma(I)]	R1 = 0.0527, wR2 = 0.1401	
R indices (all data)	R1 = 0.0610, wR2 = 0.1463	
Extinction coefficient	n/a	
Largest diff. peak and hole	0.768 and -0.654 e.$Å^{-3}$	

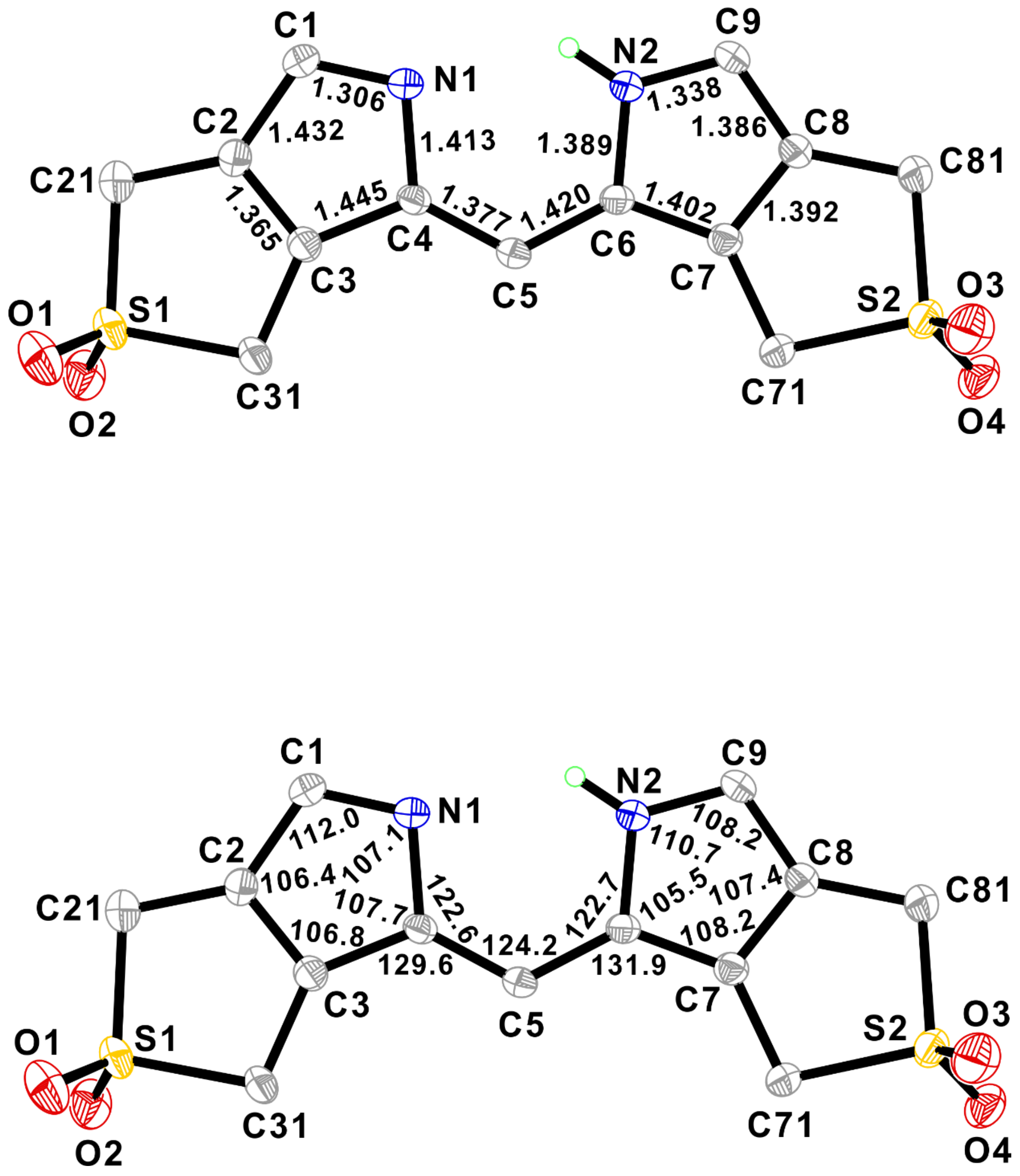

Figure S1. Front view of the dipyrrin **1** with atom numbering and relevant bond lengths (top) and bond angles (bottom). Hs from carbons and 3,5-ditert-butylphenly group were omitted for the sake of clarity.

Table S3. Crystal data and structure refinement for the di(β,β'-fullereno)pyrrin **3**.

Empirical formula	$C_{147}H_{32}N_2 \times 4CS_2$	
Formula weight	2130.26	
Temperature	193(2) K	
Wavelength	0.71073 Å	
Crystal system	Monoclinic	
Space group	C2/c (no. 15)	
Unit cell dimensions	a = 46.055(2) Å	α= 90°.
	b = 10.1641(5) Å	β= 112.763(1)°.
	c = 20.0702(8) Å	γ = 90°.
Volume	8663.3(7) $Å^3$	
Z	4	
Density (calculated)	1.633 Mg/m^3	
Absorption coefficient	0.279 mm^{-1}	
F(000)	4320	
Crystal size	0.180 x 0.150 x 0.030 mm^3	
Theta range for data collection	2.060 to 24.999°.	
Index ranges	-54<=h<=54, -12<=k<=12, -23<=l<=23	
Reflections collected	67020	
Independent reflections	7551 [R(int) = 0.0518]	
Completeness to theta = 24.999°	99.1 %	
Absorption correction	Semi-empirical from equivalents	
Max. and min. transmission	0.971 and 0.929	
Refinement method	Full-matrix least-squares on F^2	
Data / restraints / parameters	7551 / 1 / 749	
Goodness-of-fit on F^2	1.106	
Final R indices [I>2sigma(I)]	R1 = 0.0783, wR2 = 0.2042	
R indices (all data)	R1 = 0.0886, wR2 = 0.2094	
Extinction coefficient	n/a	
Largest diff. peak and hole	0.469 and -0.549 e.$Å^{-3}$	

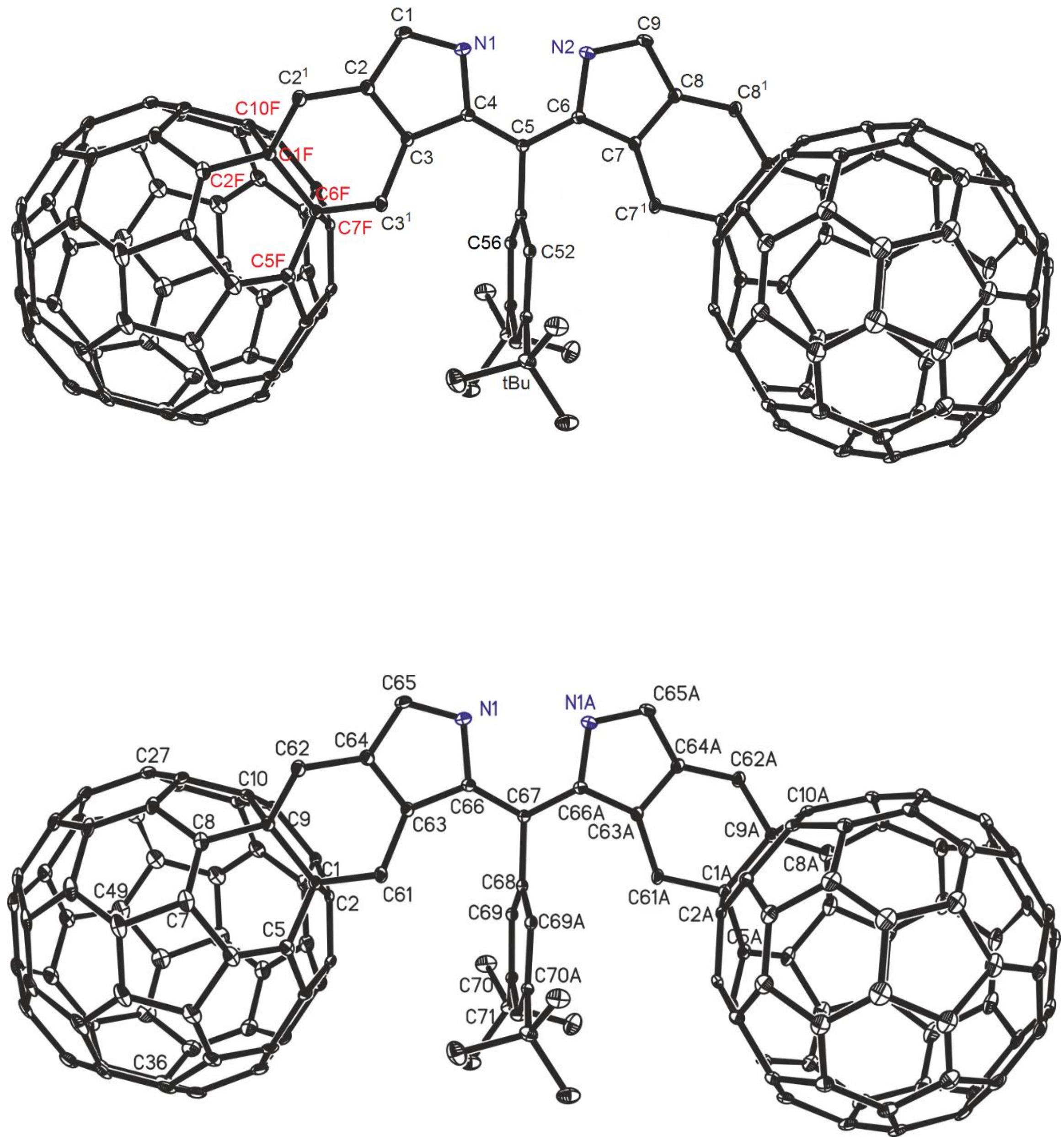

Figure S2A. Atom numbering of bisadduct **3** as used in NMR-spectroscopy (top) and in the crystal structure analysis (bottom).

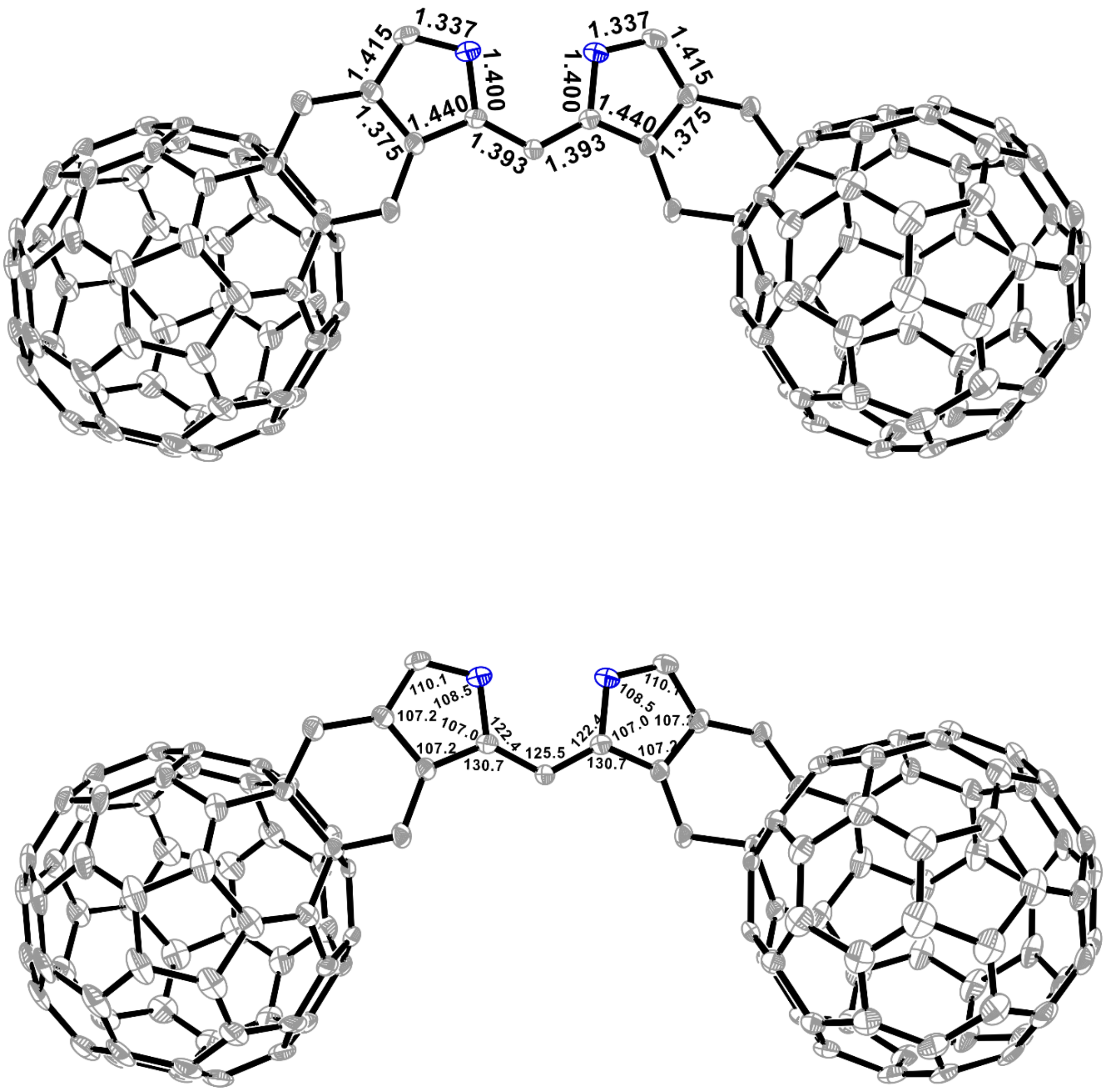

Figure S2B. Front view of the di(β,β'-fullereno)pyrrin **3** with relevant dipyrrins bond lengths (top) and bond angles (bottom). Due to the apparent symmetry of the molecule in the crystal structure the pyrrole NH is not located on either one of the two dipyrrin N-atoms. Due the crystallographic C2 symmetry (two-fold rotation axis through the atoms C67, C68 and C71, crystallographic numbering) the dipyrrin unit shows disorder by the symmetry element, giving averaged bond distances and angles in the apparently symmetry-equivalent positions. H-atoms from carbons and 3,5-di-tert-butylphenly group were omitted for the sake of clarity.

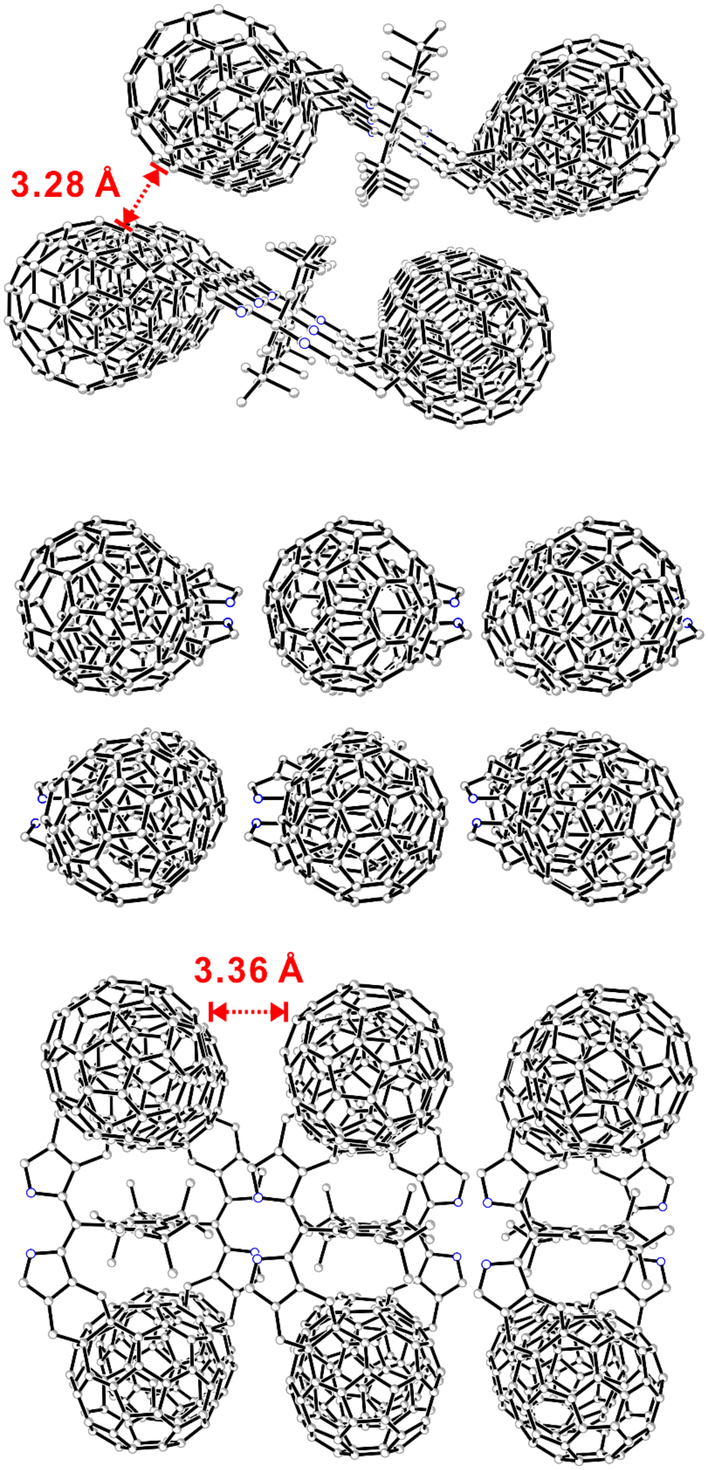

Figure S3. Crystal structure of fullerene-bisadduct **3** highlighting close intermolecular contacts and the crystal packing in projections oriented along the front, side and top faces of **3**. H-atoms were omitted for clarity.

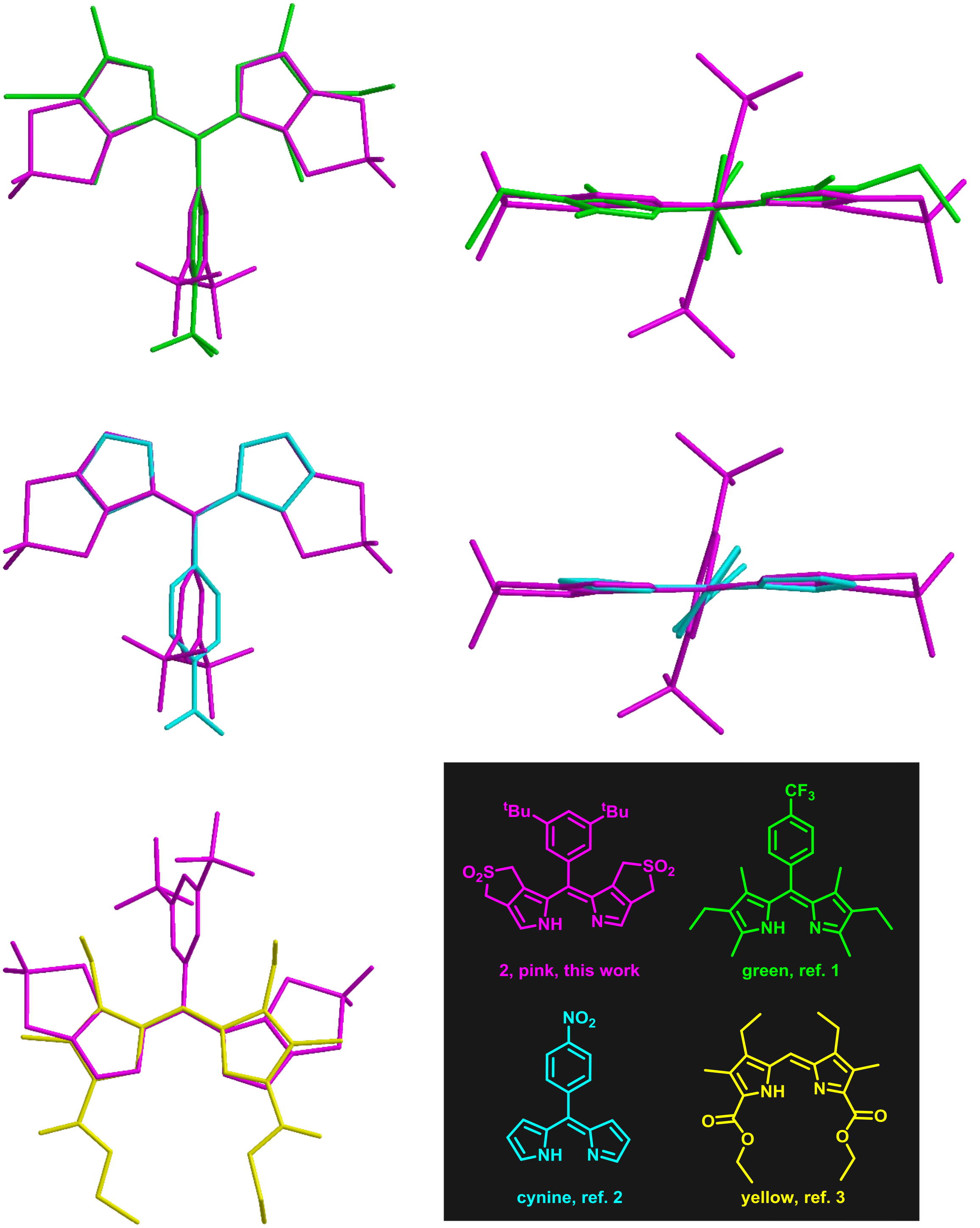

Figure S4. Comparison of crystal structures between **2** (pink) and other reported dipyrrins in difference colours. [1-3]

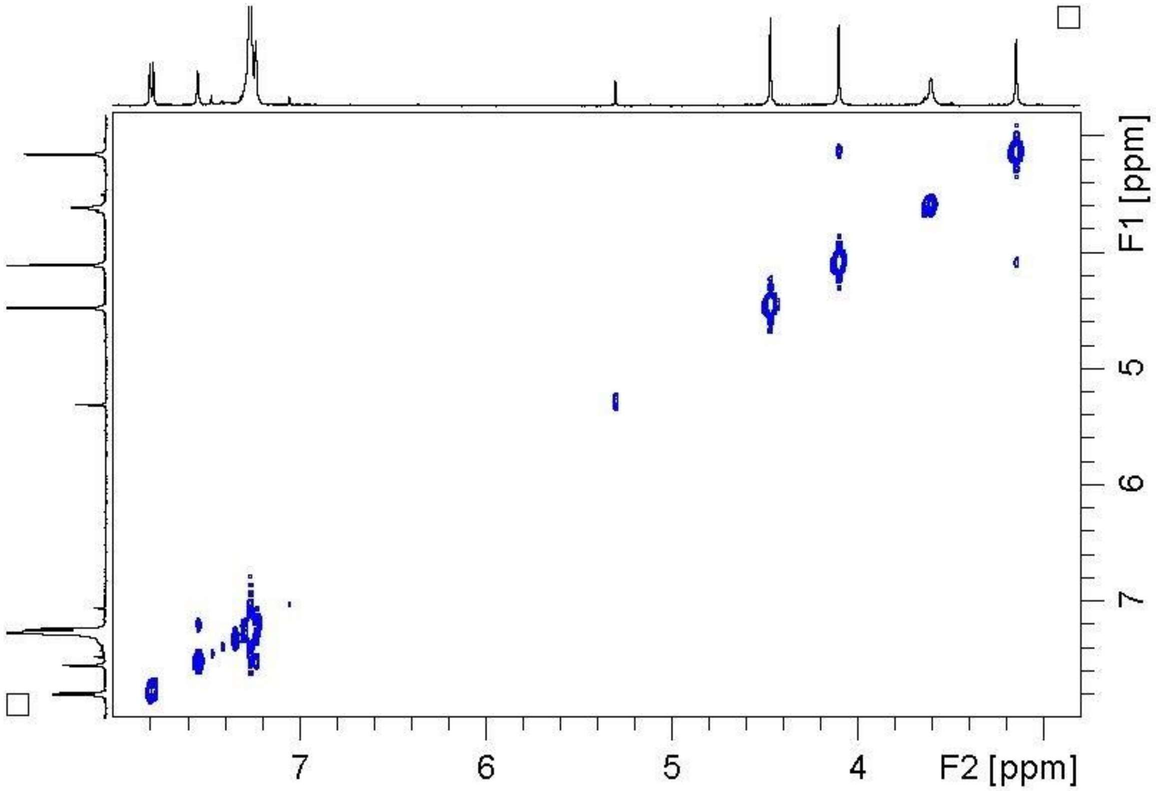

Figure S5. ^{1}H,^{1}H-COSY spectrum of mono-fullerene adduct **2** (in $CDCl_3$ / CS_2 = 4 / 1, 500MHz, 25 °C)

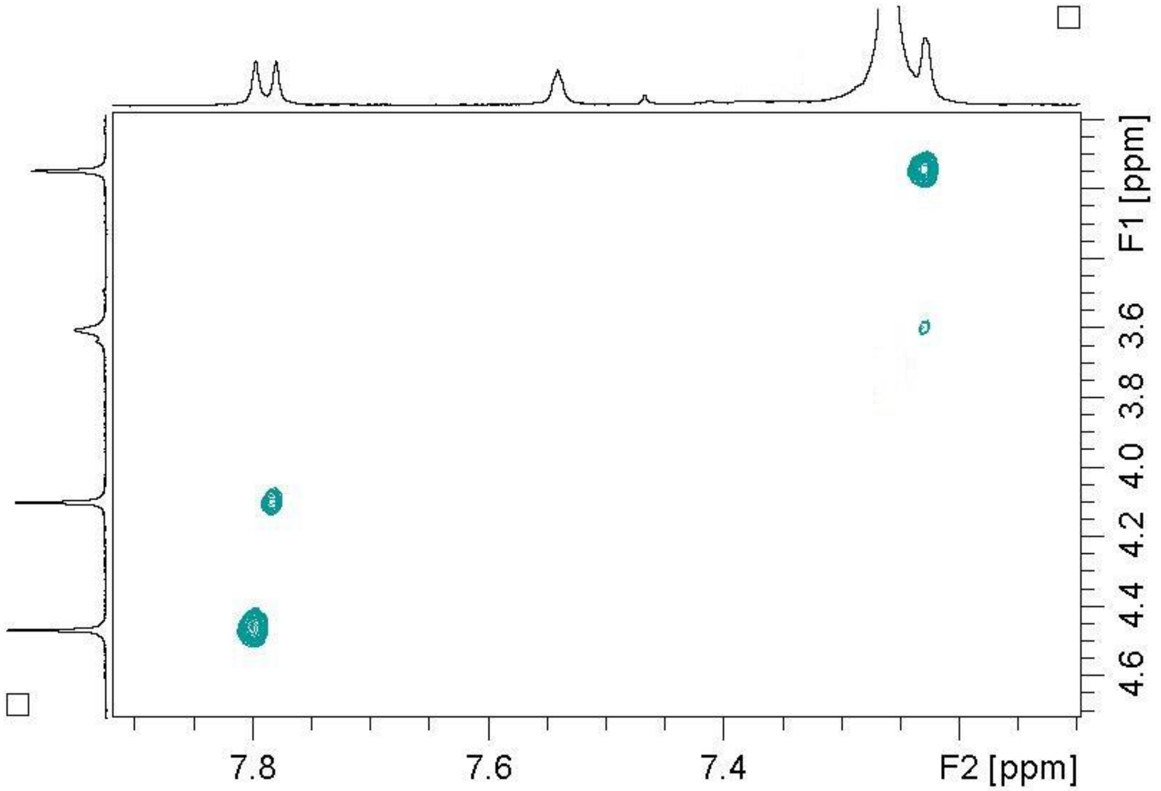

Figure S6. ^{1}H,^{1}H-ROESY spectrum of fullerene-mono-adduct **2** (in $CDCl_3$ / CS_2 = 4 / 1, 500MHz, 25 °C)

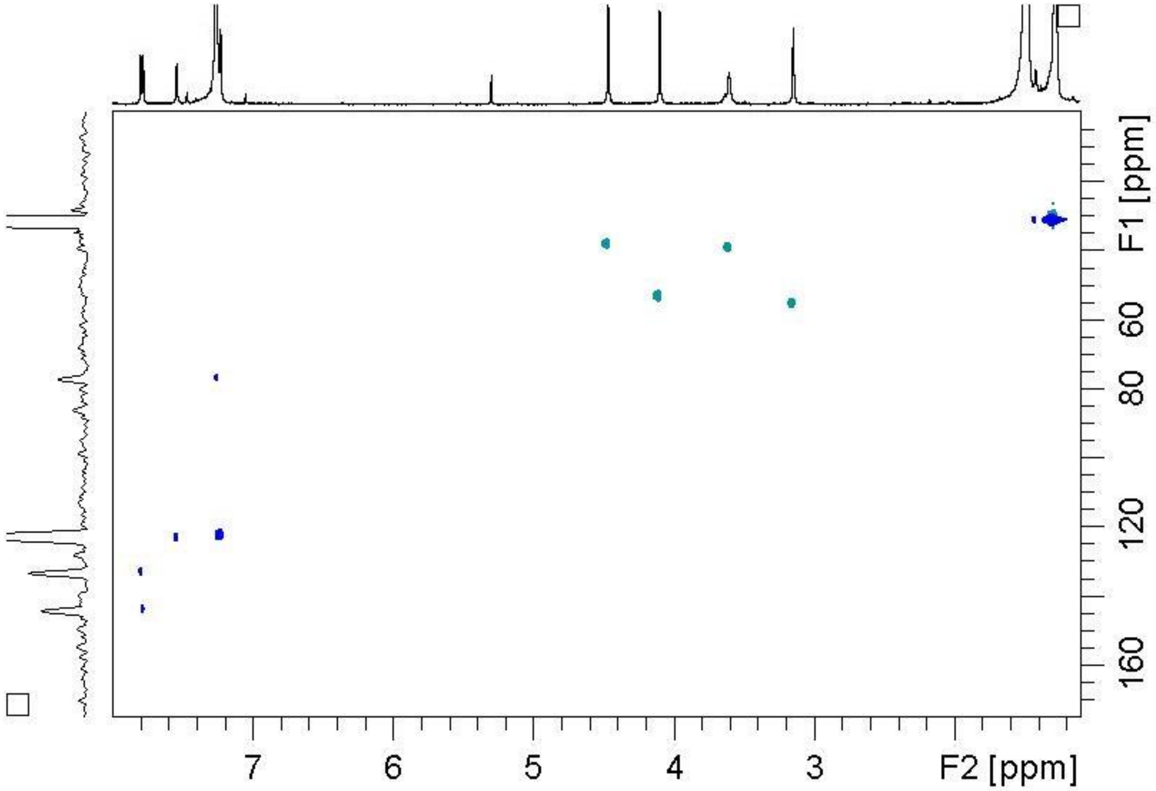

Figure S7. ^{1}H,^{13}C-HSQC spectrum of fullerene-mono-adduct **2** (in $CDCl_3$ / CS_2 = 4 / 1, 500MHz, 25 °C)

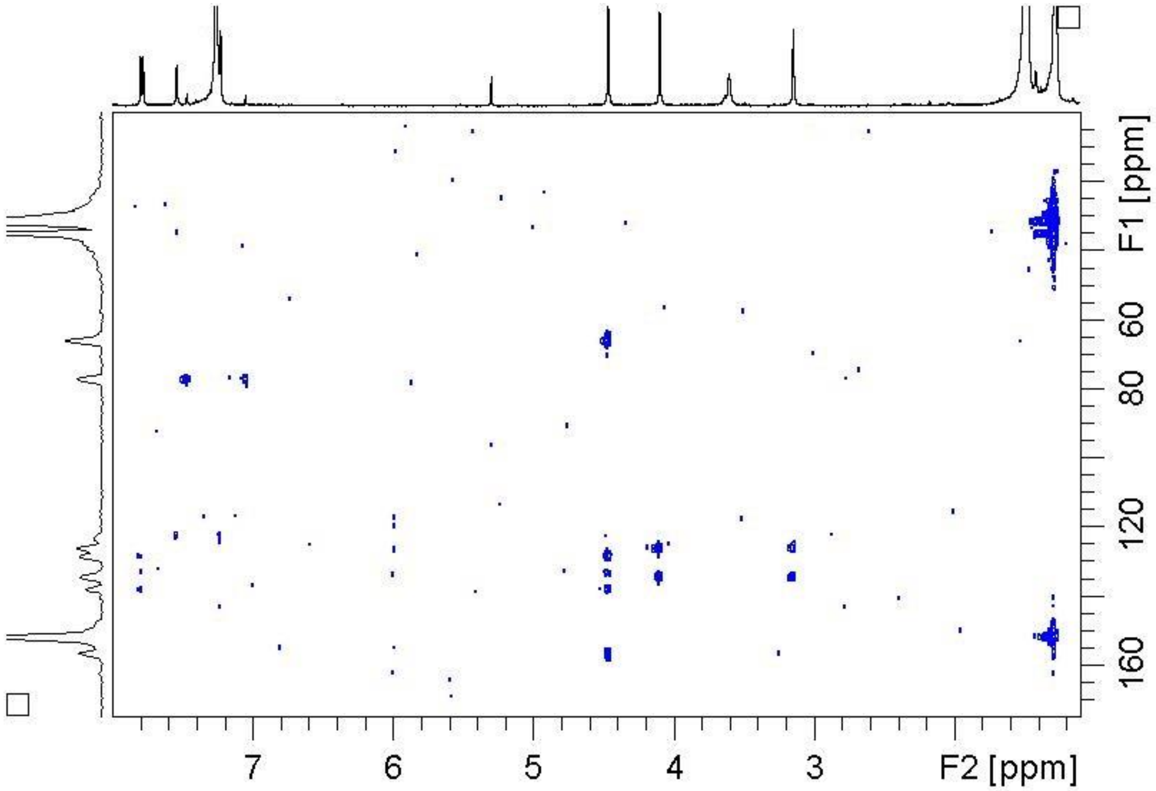

Figure S8. $^{1}H,^{13}C$-HMBC spectrum of fullerene-mono-adduct **2** (in $CDCl_3$ / CS_2 = 4 / 1, 500MHz, 25 °C)

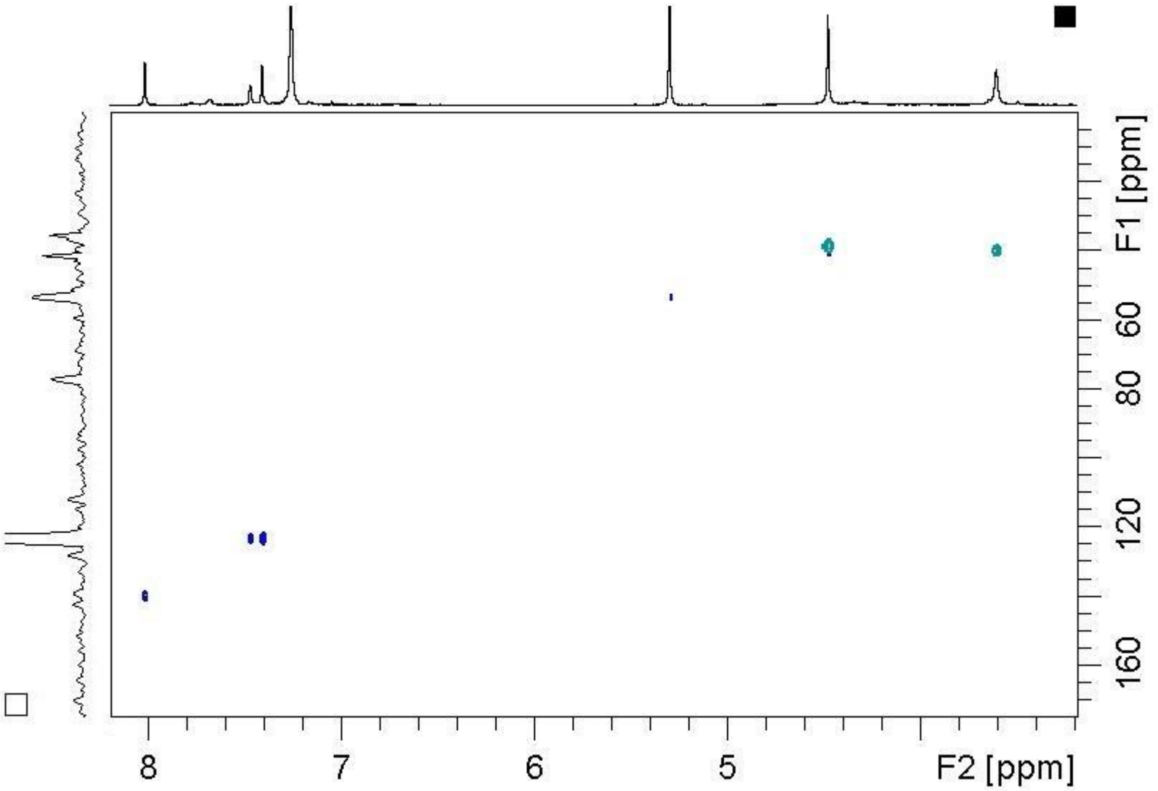

Figure S9. $^{1}H,^{13}C$-HSQC spectrum of fullerene-bis-adduct **3** (in $CDCl_3$ / CS_2 = 7 / 3, 500MHz, 25 °C)

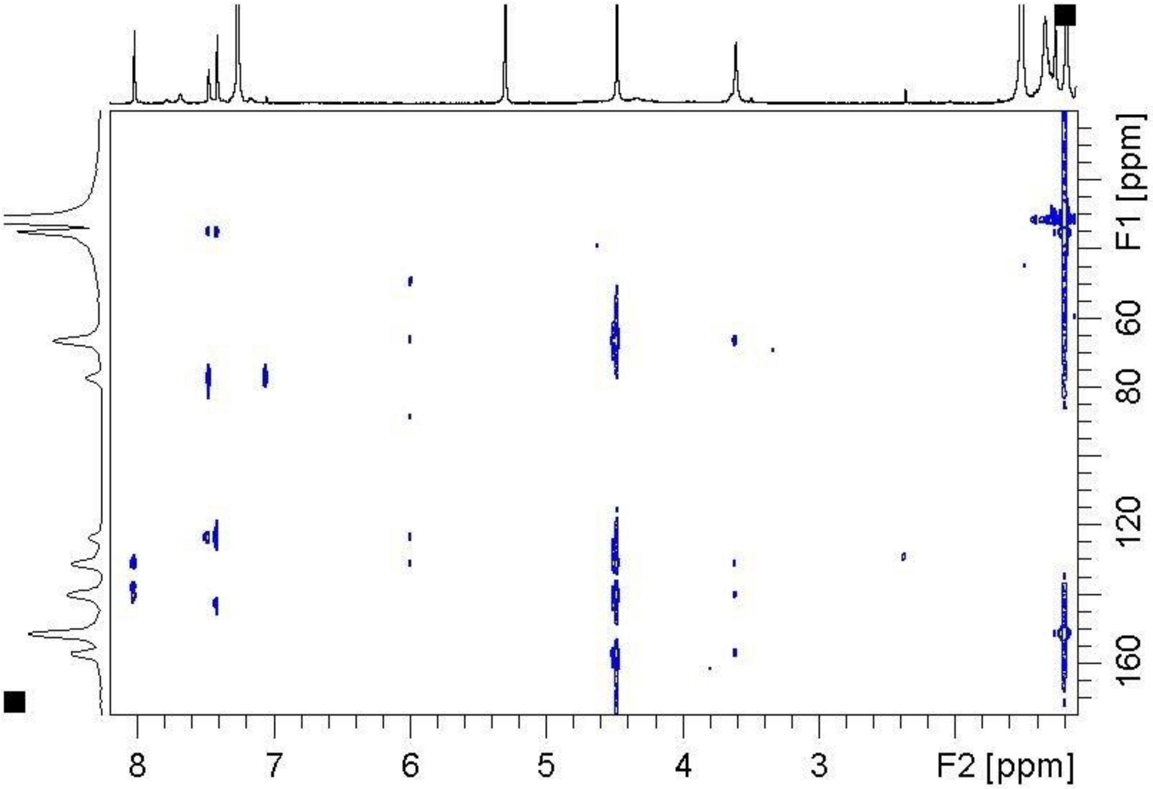

Figure S10. $^{1}H,^{13}C$-HMBC spectrum of fullerene-bis-adduct **3** (in $CDCl_3$ / CS_2 = 7 / 3, 500MHz, 25 °C)

Reference

[1] Ali, A. A.; Cipot-Wechsler, J.; Crawford, S. M.; Selim, O.; Stoddard, R. L.; Cameron, T. S.; Thompson, A. *Can. J. Chem.* **2010**, *88*, 725.

[2] Shin, J. Y.; Patrick, B. O.; Dolphin, D. *CrystEngComm* **2008**, *10*, 960.

[3] Sheldrick, W. S.; Borkenstein, A.; Stuckmeier, G.; Engel, J. *Acta. Crystallogr.* **1978**, *B**34***, 329.
